# Does Nasal Surgery Affect Right Ventricular Myocardial Functions at the Tissue Level in Patients with Nasal Septum Deviation?

**DOI:** 10.3390/jcm7080186

**Published:** 2018-07-27

**Authors:** Ziya Simsek, Eda Simsek

**Affiliations:** 1 Clinic of Cardiology, University of Health Sciences, Kayseri Education and Research Hospital, Kayseri 38100, Turkey; ziyamposta@hotmail.com; 2 Clinic of Ear, Nose and Throat, University of Health Sciences, Kayseri Education and Research Hospital, Kayseri 38100, Turkey

**Keywords:** nasal septum deviation, nasal surgery, right ventricle, myocardial function, speckle tracking echocardiography

## Abstract

Objective: One of the most common causes of upper airway obstruction in adults is nasal septum deviation (NSD). The chronic hypoxia caused by this obstruction gradually leads to increased pulmonary vascular resistance, pulmonary hypertension (PHT), and right ventricular (RV) failure. The purpose of this study was to determine changes in RV myocardial functions at the tissue level before, and after surgery in patients with NSD. Subjects and Methods: Fifty-eight patients with symptoms of nasal obstruction and snoring were included in this observational study. Preoperative and postoperative third-month peripheral arterial oxygen saturation (SpO2), and RV systolic and diastolic functions measured by pulmonary artery systolic pressure (PASP), tissue Doppler parameters, and speckle tracking echocardiography (STE) were studied in these patients. Results: We observed a very significant decrease in PASP in the postoperative period (32.54 ± 5.24 mmHg vs. 24.22 ± 4.55 mmHg, *p* = 0.001). Postoperative SpO2 values, measured at room temperature also increased significantly (93.5 ± 0.82% vs. 95.6 ± 0.79%, *p* = 0.001). There was a significant improvement after surgery in RV systolic functions, represented by global longitudinal strain (GLS) (21.12 ± 2.07 vs. 22.49 ± 1.89, *p* = 0.013) and systolic global longitudinal strain rate (GLSRs) (1.30 ± 0.12 vs. 1.38 ± 0.13, *p* = 0.015). No significant differences in terms of RV diastolic function parameters were detected, including the RV early diastolic global longitudinal strain rate (GLSRe) (1.56 ± 0.21 vs. 1.55 ± 0.26, *p* = 0.86) and RV late diastolic global longitudinal strain rate (GLSRa) (0.88 ± 0.19; 0.89 ± 0.18, *p* = 0.76). Conclusion: This study was performed with an advanced technique capable of tissue level examination. The findings demonstrated significant improvement in both chronic hypoxia and RV systolic myocardial functions, measured at the tissue level after nasal surgery.

## 1. Introduction

The nose constitutes the main conduit of the upper respiratory tract and is responsible for approximately half of airway resistance. Nasal septum deviation (NSD), is a frequent cause of nasal obstruction in adult patients. Other causes of upper airway obstruction include adenoid vegetation, tonsillar hypertrophy, nasal polyps, and nasal valve pathologies [1,2,3,4,5,6,7]. Obstruction in the upper respiratory tract reduces air flow, and causes chronic alveolar hypoxia and hypercapnia, leading to increased pulmonary vascular resistance, pulmonary hypertension (PHT), and eventually right ventricular (RV) failure [6].

The main diagnostic procedure in the evaluation of the effect of this obstruction on RV functions, is the transthorasic echocardiography. The effects of upper airway tract obstruction on the right ventricle have been evaluated using various echocardiographic methods [7,8,9,10,11]. These include measurements of pulmonary artery pressure, tissue Doppler, RV volumes, and the myocardial performance index. However, these methods have several important disadvantages, including operator dependency and the risk of being influenced by external factors [12].

The recently developed two-dimensional (2D) strain–strain rate or speckle tracking echocardiography (STE) is a new, non-invasive, reproducible, objective and minimally operator-dependent method, which measures myocardial functions at the tissue level [13]. The strain denotes the percentage of dimensional deformation in myocardial tissue, while the strain rate refers to the rate of this deformation. These methods are useful in detecting subclinical dysfunction in different pathological states [14,15].

This study, evaluated the RV functions in patients with NSD-related nasal obstruction before and after the corrective surgery, using conventional and STE parameters.

To the best of our knowledge, this was the first study using this method to evaluate patients who underwent surgery due to NSD in terms of RV function in the postoperative period.

## 2. Subjects and Methods

The study commenced post-approval, from the Erzurum Regional Training and Research Hospital Clinical Research Ethics Committee (approval No. 2016/11). All procedures involving human participants were performed in accordance with the ethical standards of the institutional and/or national research committee and were in line with the Helsinki Declaration, and its later amendments or comparable ethical standards. The study was conducted between, June 2016 and May 2017 at the Cardiology and Otorhinolaryngology departments of Kayseri Education and Research Hospital (Kayseri, Turkey).

Fifty-eight patients scheduled for septoplasty due to nasal obstruction and snoring were included in this observational study. All patients were informed about the study and they gave written their consent to participate. Anterior rhinoscopic and transnasal endoscopic examinations of all patients were assessed by an otorhinolaryngologist. Patients with marked NSD, with or without inferior concha hypertrophy were selected. All patients underwent echocardiographic evaluation before, and three months after surgery. Patients with a history of cardiovascular disease, diabetes mellitus, hypertension, sleep apnea, chronic obstructive pulmonary disease, smoking or other conditions that might cause RV dysfunction or pulmonary hypertension were excluded from the study. Other exclusions, comprised of patients with a history of long-term drug use for chronic diseases and upper airway tract obstructions other than NSD or inferior concha hypertrophy (such as middle concha and nasal valve pathologies, allergic rhinitis, nasal polyp, adenoid hypertrophy).

The peripheral oxygen saturation (SpO2), heart rate, diastolic blood pressure (DBP) and systolic blood pressure (SBP) of all patients, were measured using a pulse oximeter in the same room before, and three months after surgery. Body weight and height were also recorded. Body mass index (BMI) was calculated as weight in kilograms divided by the square of height in meters (kg/m^2^).

Nasal obstruction levels were determined using the Nose Obstruction Symptom Evaluation (NOSE) scale. Patients with NOSE scores of 75–100 were included in the study [16,17].

## 3. Surgical Procedure

All patients were operated on by the same surgeon, under general anesthesia. A standard septoplasty technique was applied in all cases. Each nasal cavity was packed with a piece of gauze pack, containing adrenaline dissolved in saline at a concentration of 1:200,000. The gauze was removed 15 min after the start of surgery. The cartilaginous and bony nasal septum was exposed from both sides by the elevation of mucoperichondrial and mucoperiosteal flaps. Deviated structures were then removed [18]. Sufficient cartilage and bone were preserved, and inferior concha surgery was performed for those patients requiring it. Internal nasal splints were installed at the end of surgery and removed on the postoperative second day. All patients were discharged on the second postoperative day. Cases were reviewed after one week, and in the first and third months after surgery.

## 4. Echocardiography

Echocardiographic measurements of all patients were performed using a 2.5 MHz probe and GE Vingmed Vivid 7 echocardiography device (GE Ultrasound, Horten, Norway), under electrocardiography (ECG) monitoring, with patients in the left lateral decubitus position, as recommended by the American Society of Echocardiography guidelines [19]. All measurements were recorded on a digital workstation (Echopac Workstation; Vingmend Ultrasound GE) for offline analysis. Left ventricular ejection fraction (LVEF) was measured using the Simpson method. Left ventricular end systolic diameter (LVESD), left ventricular end diastolic diameter (LVEDD), left atrial (LA) diameter, and interventricular septum diastolic (IVSd) thickness were measured using M-mode echocardiography in parasternal long axis views. Right atrial end diastolic (RAED) and right ventricular end diastolic (RVED) diameters were measured from apical four chamber images. Pulmonary artery systolic pressure (PASP), was calculated with the simplified Bernoulli equation using tricuspid flow velocity, and addition of right atrial pressure (mean 5 mmHg) estimated based on the respiratory collapse of the inferior vena cava. Systolic (Sm), early diastolic (Em) and late diastolic (Am) tissue Doppler measurements were taken from apical four chamber images by activating the device’s velocity integral (TVI) function.

## 5. Speckle Tracking Echocardiography

**Figure 1:**
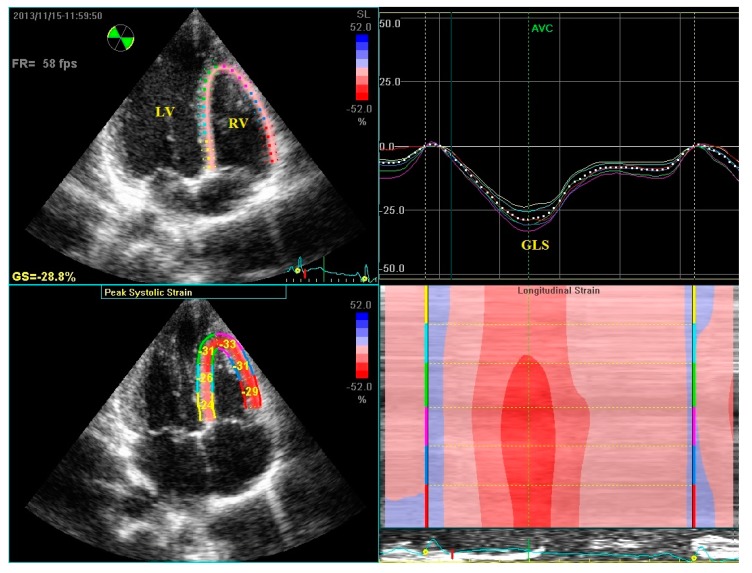
Example of global systolic strain (GLS) in the right ventricle from apical four chamber view. RV: right ventricle, LV: left ventricle.

RV images were taken from apical four chamber images. Three consecutive cycles in deep inspiration were recorded. The images with the best visible RV endocardial margins were then selected. Recorded images were transferred to the workstation (EchoPAC, GE Vingmed Ultrasound) for offline analysis. The frame rate was 50–90 frames/s. The automated function imaging (AFI) protocol was applied for strain and strain rate analyses. A mean six to seven points were selected starting from the basal septum. After shifting to the cardiac cycle dynamic mode of the selected image, the parts of the selected points out of the pericardium were corrected manually. The consistency of the selected image with aortic valve closure time, and end of the electrocardiographic T wave was then checked. Global longitudinal systolic strain (GLS), global longitudinal systolic strain rate (GLSRs), global longitudinal early diastolic strain rate (GLSRe) and the global longitudinal late diastolic strain rate (GLSRa) values, were measured from each image [20,21] (Figure 1).

## 6. Statistical Analysis

Continuous variables were presented as mean ± standard deviation, and categorical variables as percentages. The Kolmogorov-Smirnov test was used for analysis of distribution of variables. All continuous variables exhibited normal distribution. The paired samples *t* test, was used to compare parametric variables between pre- and postoperative third month measurements. SPSS 19.0 statistical analysis software (SPSS Inc., Chicago, IL, USA) was used to evaluate and test variables. *p* values < 0.05 were considered significant.

## 7. Results

**Figure 2:**
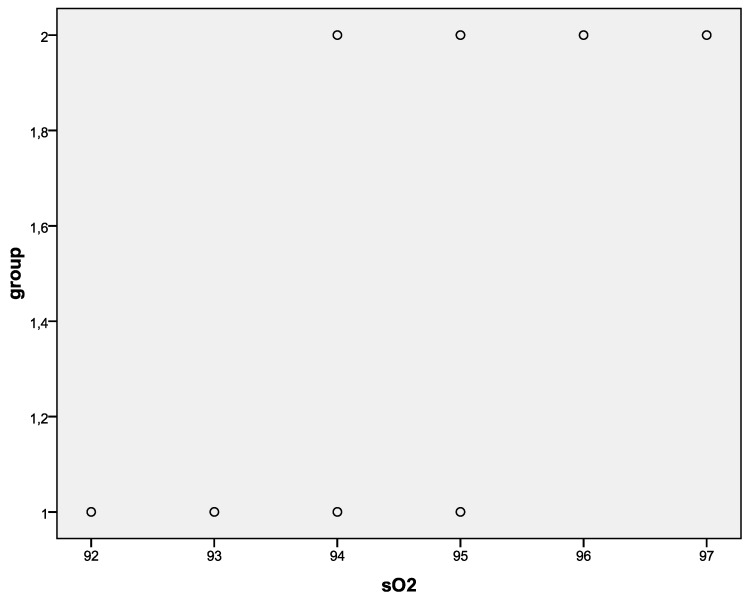
Scatter graph of preoperative and postoperative SpO2 values Group 1—Preoperative SpO2 values, Group 2—postoperative SpO2 values.

Patients’ demographic data are shown in Table 1. The mean age of the 58 patients in the study was 36 ± 7 years; where, 38 (65%) were male and 20 (35%) were female. A highly statistically significant improvement was noted in the patients’ mean NOSE scores (80.78 ± 8.13; 20.47 ± 6.12, *p* < 0.01). Preoperative and postoperative third month echocardiographic and oxygen saturation data, are shown in Table 2. LV diameters and functions were similar at pre and postoperative measurements. RV and right atrial diameters exhibited no difference in the postoperative period. A statistically highly significant decrease in PASP in the postoperative period (32.54 ± 5.24 mmHg vs. 24.22 ± 4.55 mmHg, *p* = 0.001) was observed. A significant increase was also determined in postoperative SpO2 values, measured at room temperature (93.5 ± 0.82% vs. 95.6 ± 0.79%, *p* = 0.001) (Figure 2). Tissue Doppler and global strain-strain rate data are presented in Table 3. Tissue Doppler systolic (Sm) and diastolic (Em, Am) parameters measured from the tricuspid lateral annulus were similar in the pre and postoperative assessments. There was a significant postoperative improvement in RV systolic function, represented by GLS (21.12 ± 2.07 vs. 22.49 ± 1.89, *p* = 0.013) and GLSRs (1.30 ± 0.12 vs. 1.38 ± 0.13, *p* = 0.015). We observed no significant difference, in terms of RV diastolic function parameters including RV GLSRe (1.56 ± 0.21 vs. 1.55 ± 0.26, *p* = 0.86) and RV GLSRa (0.88 ± 0.19 vs. 0.89 ± 0.18, *p* = 0.76) (Table 3).

## 8. Discussion

NSD and concha hypertrophy, are frequent causes of nasal obstruction in patients who snore. Other causes of upper airway obstruction include adenoid vegetation, tonsillar hypertrophy, nasal polyps, allergic rhinitis, and nasal valve pathologies. Several studies have examined the adverse effects of NSD on the heart [1,2]. Recent research on the subject has shown that upper respiratory tract obstruction causes chronic alveolar hypoxia and hypercapnia, leading to PHT and RV failure. Previous studies have also reported that RV functions recovered, and pulmonary artery pressure decreased significantly after surgical correction of upper airway obstruction in patients with adenotonsillar hypertrophy, NSD and similar pathologies [1,2,3,4,5,6,7,8,9,10]. This study found a significant postoperative improvement in RV systolic functions at the tissue level in NSD patients. Mean PASP increased (from 32.54 ± 5.24 mmHg to 24.22 ± 4.55 mmHg), whilst SpO2 values decreased (from 93.5 ± 0.82% to 95.6 ± 0.79%) among patients with NSD. There was also a significant postoperative improvement in RV systolic function, represented by GLS (from 21.12 ± 2.07 to 22.49 ± 1.89) and GLSRs (from 1.30 ± 0.12 to 1.38 ± 0.13). These results are compatible with those of previous studies. The factor that particularly differentiates this study from other research, is that the echocardiographic method employed was more objective.

Chronic hypoxia due to obstruction in the upper respiratory tract, such as NSD, induces pulmonary vasoconstriction and leads to increased pulmonary capillary pressure. This increase in pulmonary capillary pressure can result in PHT and RV failure [6,22]. PHT increases RV pressure and causes RV hypertrophy, dilatation and finally, RV dysfunction [23]. There is usually a lengthy asymptomatic period until RV dysfunction occurs [24]. Once the patient has become symptomatic, the process is generally irreversible. Early assessment of RV functions is therefore crucial in patients with upper respiratory tract obstruction, and early surgery can prevent potentially hazardous effects in patients with signs of RV failure. Once the pathology leading to upper airway obstruction has been relieved, chronic alveolar hypoxia, hypoxic pulmonary vasospasm and pulmonary vascular resistance decrease. SpO2 at room temperature in a healthy individual is approximately 97% [25]. In this study group, the mean preoperative and postoperative third-month SpO2 values were 93.5 ± 0.82% and 95.6 ± 0.79%, respectively. This showed that the chronic hypoxia in the patients recovered to a near-normal value.

Many parameters, such as PASP, tricuspid annular plane systolic excursion (TAPSE), fractional area change (FAC), RV index of myocardial performance (RIMP), and right isovolumic myocardial acceleration (IVA) can be used to evaluate RV systolic functions. However, these parameters are volumetric load—and angle-dependent [26]. In routine practice, RV failure is most commonly evaluated using PASP. The mean preoperative PASP value in the study group was 32.54 ± 5.24 mmHg, indicating mild PHT [27]. PASP decreased significantly to 24.22 ± 4.55 mmHg in the postoperative third month. Various studies have reported a decrease in PASP together with recovery of RV functions, measured using different echocardiographic methods after treatment of NSD or an upper airway tract obstruction [1,2,7,8,9,10]. The findings of this study, were compatible with those of previous studies. However, scant data are available in those studies regarding the effect of the RV function recovery on myocardial tissue. One superior aspect of this study was that the echocardiographic method employed revealed changes at the tissue level.

The evaluation of RV functions is challenging because of the complex structure in patients with mild PHT. The principal method for determining global or segmental RV functions is tissue Doppler imaging. However, this also poses several disadvantages, including active and passive tracking effects of the adjacent myocardial tissues and angle dependence. STE is a tissue Doppler-based technique capable of measuring both systolic and diastolic functions without angle and with minimal operator dependence. It has been described as a more suitable echocardiographic method for the assessment of RV functions [13,28]. RV longitudinal strain is a non-invasive marker of RV contractility, which is not affected by complex RV geometry and is independent of volume load [29]. A study of 575 patients with PHT was conducted in Reference [30], identified RV longitudinal strain as a strong predictor of RV function, well correlated with the objective clinical functional class defined by the World Health Organization, the six-minute walk test, and the NT-proBNP mediator. Additionally, deteriorated RV strain has been linked to numbers of hospitalizations and all-cause mortality [30].

The automated function imaging (AFI) protocol was originally designed for LV strain analysis. However, an assessment of global and regional strain values using AFI has been shown to be superior to 2D strain and more sensitive to RV pressure loadings [20]. This study, observed no significant change in tricuspid lateral annular systolic and diastolic velocities, measured using tissue Doppler imaging. Diastolic strain and strain rate values also exhibited no differences. However, GLS and GLSRs values, which indicated RV systolic functions, recovered significantly in the postoperative period. No signs or symptoms of clinical RV failure were observed in the study group. Significant subclinical improvement was determined in systolic myocardial functions of the right ventricle.

Study limitations: STE-based imaging necessitates better image quality and clear definition of endocardial borders. This study excluded patients with poor image quality. Acoustic rhinometry measurements could have also provide more objective results, but this was not possible citing technical reasons.

## 9. Conclusions

Our study’s findings indicated significant improvement after surgery in both chronic hypoxia, and RV systolic myocardial functions measured at the tissue level in patients with marked NSD.

Nasal obstruction has subclinical effects on the right ventricle, which is a cause of significant health problems. Therefore, patients who need septoplasty operations should be informed about the procedure’s positive effects on the cardiac system. Clinicians should be aware that correction of NSD has the potential to improve the RV systolic function and should consider early septoplasty to avoid the development of RV systolic dysfunction and heart failure.

## Figures and Tables

**Table 1:** Demographic data of patients with nasal septal deviation.

*n*	58
Age, years	36 ± 7
Male, *n* (%)	38 (65)
Female, *n* (%)	20 (35)
BMI, kg/m^2^	22.8 ± 2.1
Heart rate, beats/min	74.2 ± 6.6
Systolic blood pressure, mmHg	118 ± 14
Diastolic blood pressure, mmHg	69 ± 8

BMI—body mass index, DBP—diastolic blood pressure, SBP—systolic blood pressure.

**Table 2:** Pre- and postoperative echocardiographic and oxygen saturation data.

	Preoperative	Postoperative Third Month	*p* *
LVED, mm	48.21 ± 1.96	49.20 ± 2.12	0.88
LVES, mm	29.92 ± 1.56	30.85 ± 1.82	0.72
LA, mm	32.22 ± 1.19	32.56 ± 1.25	0.94
IVSd, mm	8.93 ± 1.18	8.84 ± 1.15	0.78
LVEF, Simpson %	65.9 ± 2,31	66.1 ± 2.44	0.96
RVED, mm	33.1 ± 2.21	32.8 ± 2.1	0.80
RAED, mm	32.8 ± 2.16	32.1 ± 2.34	0.78
SpO2, %	93.5 ± 0.82	95.6 ± 0.79	0.001
PASP, mmHg	32.54 ± 5.24	24.22 ± 4.55	0.001

Values are expressed as mean ± standard deviation and number (percentage), * Paired samples *t* test for continuous variables. IVSd-inter ventricular septum end diastolic diameter, LA—left atrium, LVEF—left ventricle ejection fraction, LVED—left ventricle end-diastolic diameter, LVES—left ventricle end-systolic diameter, RAED—right atrium end-diastolic diameter, RVED—right ventricle end-diastolic diameter, PASP—pulmonary arterial systolic pressure, SpO2—peripheral arterial oxygen saturation.

**Table 3:** Pre- and postoperative tissue Doppler and global strain–strain rate data.

	Preoperative	Postoperative Third Month	*p* *
Tricuspid lateral annulus Sm, cm/s	13.11 ± 1.34	13.79 ± 1.12	0.51
Tricuspid lateral annulus Em, cm/s	14.6 ± 2.1	15.1 ± 2.7	0.48
Tricuspid lateral annulus Am, cm/s	11.3 ± 3.49	11.3 ± 3.43	0.96
RV GLS, %	21.12 ± 2.07	22.49 ± 1.89	0.013
RV GLSRs, 1/sec	1.30 ± 0.12	1.38 ± 0.13	0.015
RV GLSRe, 1/sec	1.56 ± 0.21	1.55 ± 0.26	0.86
RV GLSRa, 1/sec	0.88 ± 0.19	0.89 ± 0.18	0.76

Values are expressed as mean ± standard deviation and number (percentage), * Paired samples *t* test for continuous variables. Am—late diastolic myocardial velocity, Em—early diastolic myocardial velocity, GLS—global longitudinal systolic strain, GLSRs—global longitudinal systolic strain rate, GLSRe—global early diastolic strain rate, GLSRa—global late diastolic strain rate, RV—right ventricle, Sm-systolic myocardial velocity.
